# I can’t count, but I can beat you playing cards: a case report on autoimmune encephalitis

**DOI:** 10.1186/s12883-021-02370-x

**Published:** 2021-09-10

**Authors:** Laura Mori, William Campanella, Lucilla Vestito, Lucio Marinelli, Luana Benedetti, Leonardo Cocito, Carlo Trompetto

**Affiliations:** 1 Department of Neuroscience, Rehabilitation, Ophthalmology, Genetics, Maternal and Child Health, University of Genoa, Genoa, Italy; 2 IRCCS Ospedale Policlinico San Martino, Genoa, Italy

**Keywords:** Autoimmune limbic encephalitis, Cognitive dysfunction, Dyscalculia, Single-case

## Abstract

**Background:**

Autoimmune encephalitis (AE) is a rare inflammatory disorder characterized by important psychiatric and neurologic symptoms. The literature documents high rates of neuropsychological dysfunction in N-methyl D-aspartate-receptor (NMDAr) encephalitis but papers don’t consider specifically calculation disturbances between the long-term deficits, although deficits in executive control and episodic memory were less likely to resolve.

**Case report:**

Here we present a severe case of NMDAr encephalitis in a young patient without a relevant past medical history. Upon first examination he presented psycho-motor slowdown, speech disorders, severe cognitive deficits in all areas: concentration, attention, memory, language, dual task functions, increased latency in responses, severe dyscalculia. Upon first evaluation, the young patient underwent a battery of neuropsychological tests and he showed a dysexecutive syndrome with performances significantly low for age and education. Our patient hence underwent 1 month of intensive cognitive rehabilitation. After the rehabilitation treatment, he presented an amelioration in all domains except calculations.

**Conclusions:**

In our patient the calculation disorder has proved to be the most relevant problem and the most difficult to treat. Clinicians should consider a careful approach to determine the prognosis of this syndrome because of the wide range of deficits, the need of prolonged treatment and the rate of long-term sequelae.

## Background

Autoimmune encephalitis (AE) is an inflammatory multi-stage illness with psychiatric symptoms, seizures, cognitive impairment, movement disorders and autonomic instability. At present, one of the most common immune-mediated subtypes of AE associated with antibodies against neuronal cell surface antigens is N-methyl D-aspartate-receptor (NMDAr) encephalitis. Its pathogenesis is mediated by immunoglobulin G antibodies against the GluN1 subunit of the neuronal NMDAr (NMDAr antibodies), which determine a potentially permanent neuronal damage if untreated, due to prolonged inflammation and glutamate excitotoxicity [1].

Approximately 70% of patients have prodromal symptoms. Within a couple of weeks at the latest, patients begin to show important psychiatric symptoms, often accompanied by cognitive-behavioral disorders, confusion and disorientation. Attention and short-term memory deficits are common, but not immediately noticeable, because psychiatric and language disorders often interfere with memory evaluation [2, 3]. Focal, generalized seizures, even status epilepticus may occur early or later during the natural history of the disease [3]. Characteristic movement disorders are associated with the neuro-psychiatric symptoms and have been variably described as dyskinetic or choreo-athetoid involving face, limbs, and trunk. The clinical picture may evolve and get complicated by dysregulation of autonomic functions, requiring admission within Intensive Care Units (ICU) [3]. The evaluation of presenting symptoms and the evolution of the clinical features disclose a diffuse encephalopathy due to dysfunction of subcortical structures, limbic regions, amygdalae, and frontostriatal circuitry [2].

Many experts widely agree that well-timed diagnosis and a multi-disciplinary approach are essential for a better prognosis [4, 5]. About 75% of patients with NMDAr antibodies recover to premorbid status or have mild sequelae if promptly and adequately treated; all other patients remain severely disabled, while die is uncommon [2].

## Case presentation

**Fig. 1:**
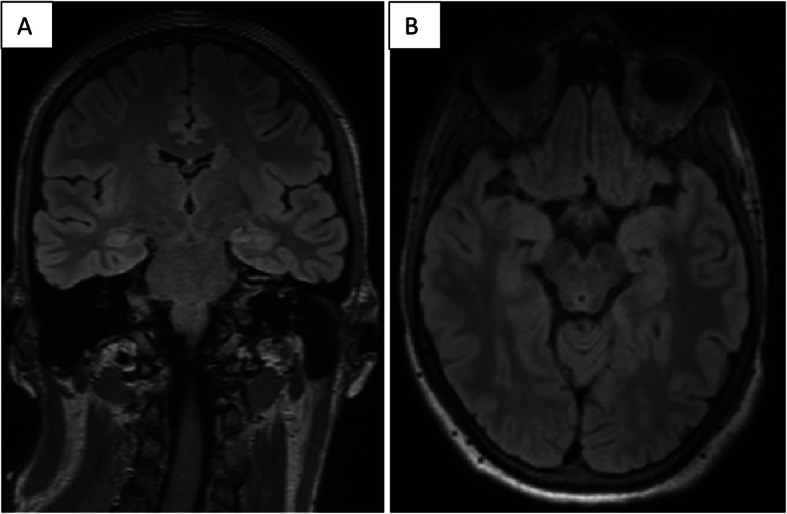
Coronal (**A**) and axial (**B**) FLAIR MRI sequences. No lesions could be disclosed during the acute phase nor during follow-up

**Fig. 2:**
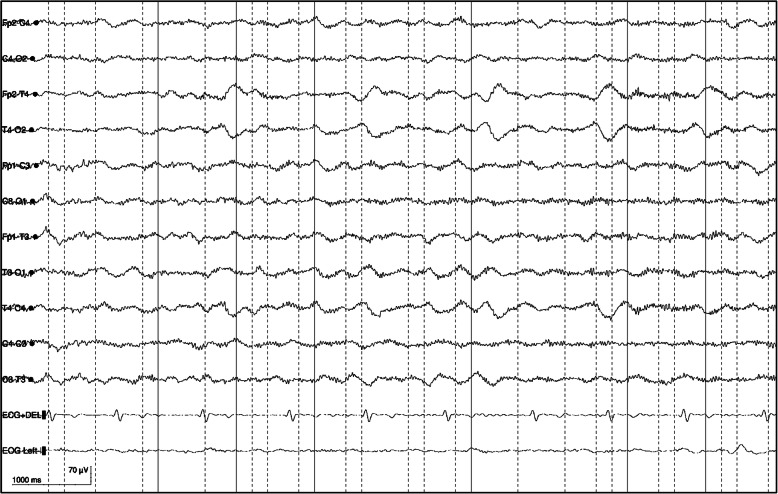
EEG performed during the acute phase. Electroencephalography performed during the acute phase before admission to the rehabilitative ward, showing typical extreme delta brush pattern. 30 mm/sec, 100 μV/cm, low pass filter 70 Hz, high pass filter 1.6 Hz, notch filter 50 Hz

Here we present a case of anti-NMDAr encephalitis in a 17-year-old male, without a relevant past medical history. He was in good health, regularly attending the fourth year of high school, and was also practicing golf competitively. He was referred to the hospital emergency room because of a partial motor epileptic seizure without loss of consciousness. The results of electroencephalography (EEG) was unremarkable and the diagnostic hypothesis of a somatoform disorder was advanced. In the following 2 months he experienced psychic deterioration, repeated episodes of loss of consciousness and epileptic seizures, language deterioration until mutism, facial and upper limbs dyskinesia, psychomotor agitation and hyperpyrexia. He was then referred to the ICU of San Martino Polyclinic Hospital-IRCCS in Genoa because of hyperpyrexia and focal motor seizures on the left side of the body with secondary generalization and status epilepticus. At the admission, the patient was sedated with propofol in order to obtain burst suppression, curarized and mechanically ventilated. Brain computed tomography (CT) and Magnetic Resonance Imaging (MRI) were performed with normal results (Fig. 1). The cerebral PET reveled hypometabolims due to sedation. There were no remarkable findings from serum sample, virology and bacterial cultures were negative. Paraneoplastic antibodies research resulted negative. Whole-body CT and PET/CT had been executed and normal scans obtained. The cerebro-spinal-fluid (CSF) researches for autoimmune antibodies revealed the presence of anti-NMDA antibodies with pleocytosis (165 cell/mm3). An EEG was repeated (Fig. 2) and, based on these clinical and paraclinical findings, the patient was diagnosed with anti-NMDAr limbic encephalitis [6] and underwent a first-line immuno-therapy with high dose intra-venous (IV) steroid (6-methylprednisolone, 1000 mg / day for 5 days) and subsequent oral steroid therapy (prednisone, 50 mg / day) for 30 days with gradual tapering. Moreover, just after IV steroid therapy, a treatment with IV immunoglobulins was started (0.4 g / kg / day for 5 days, once per month for 6 months). One month after, the patient was transferred to the neurologic unit, and, afterwards, the patient arrived to our Neuro-rehabilitation Unit to undergo intensive rehabilitative treatment. Here, upon first examination (T0), psycho-motor slowdown, speech disorders, severe cognitive deficits in all areas (concentration, attention, memory, language, dual task functions, increased latency in responses, severe dyscalculia) were found. Specifically, his spontaneous speech was non fluent, with several semantic and lexical abnormalities. He showed severe difficulties in verbalizing any personal information beyond their own name, surname and the name of the family members. Facial mimics and gestures were almost absent. Auditory and written comprehension was sufficient. He then underwent a battery of neuropsychological tests with regard to the following domains: global cognitive status (Mini-Mental State Examination (MMSE)), Simple and selective attention with visual inspection (Trail Making Test part A (TMT-A) and part B (TMT-B), Visual Search test), Phonological (p, f, l) and Semantic (animals, fruit, car brands) Fluences; Frontal Assessment Battery (FAB), Colored Progressive Matrices of Raven, Short and long-term verbal and visual episodic memory (Babcock Story Recall Test, Rey-Osterrieth Figure Copy and Recall), visuo-constructional praxis (Clock Drawing Test) (Table 1).

**Table 1:** Global cognitive evaluation

Task	Raw score T0	Raw score T1	Cut-off
**Global cognitive status**			
Mini Mental State Examination	17	24	≥24
**Attention**			
Trail Making-A	96	53	≤67
Trail Making-B	–	194	≤184
Trail Making B-A	–	1	≤107
Visual Search	22	56	≥30
**Verbal and visual memory**			
Babcock story recall test	5.5	9	≥8
Corsi block forward task (8 blocks)	6.98	6.98	≥5.75
Rey Complex Figure Test (recall)	12	16	≥4.69
**Visuo-spatial function**			
Clock Drawing Test	4	10	≤8
Rey Complex Figure Test (coping)	20.5	29	≥28.56
**Executive functions**			
Colored Raven’s Progressive Matrices (1947)	23	26	≥18.96
Frontal Assessment Battery	10	16	≥13.5
**Language**			
Verbal fluency (letter)	5	18	≥17
Verbal fluency (category)	11	33	≥25

**Fig. 3:**
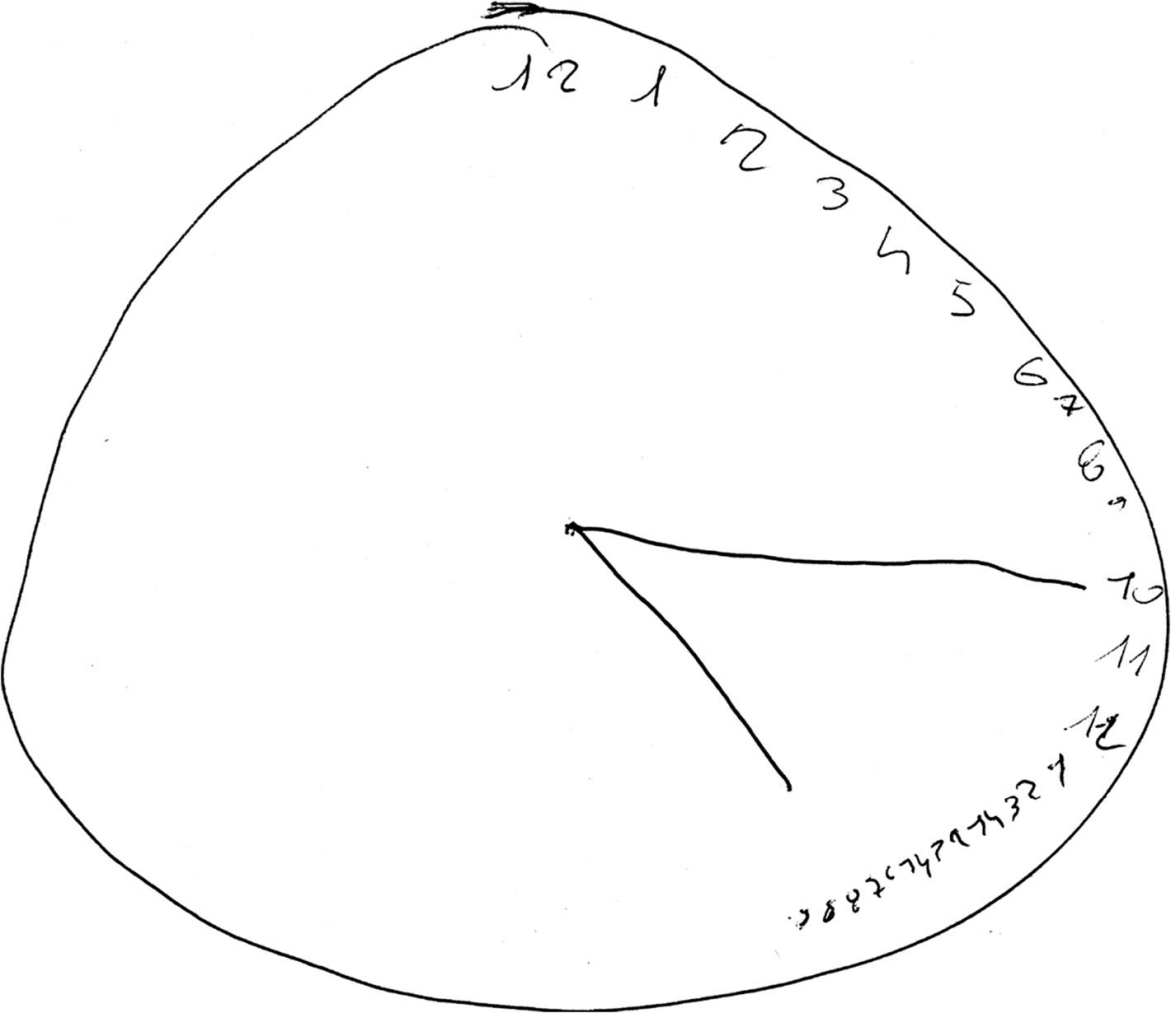
Clock Drawing Test at T0

His performances were significantly low for his age and education. At the clock drawing test (Fig. 3) he showed impaired visuo-spatial functions with perseveration, typical aspects of a dysexecutive syndrome. Language functions were evaluated with the Italian version of the Aachener Aphasia Test (AAT). His spontaneous speech was non-fluent. He was able to name only objects with high frequency of use, while he failed to name low frequency objects. Colors were correctly named. Auditory and written comprehension was sufficient.

The patient’s cognitive evaluation was completed by testing number semantic and calculation. Number semantic was tested with the following tasks: naming general and personal numerical facts (e.g. how many days are there in a year? When is it Christmas?); naming the larger of two different numbers by saying “first” or “second”; ordering dot cards by magnitude; ordering numbers by magnitude on a vertical line. Performances were correct, showing a full understanding of the numerical meaning of numbers. Calculation was investigated with single-digit calculation (12for each arithmetical operation), by both visual and auditory stimulation with spoken and written responses and multi-digit calculation (25 for each arithmetical operation) by visual stimulation with written response. In the single-digit calculation subtests, his errors were mainly non-response or long latency times and difficulties in the correct interpretation of the arithmetical signs. In the multi-digit calculation, the types of error were mostly the syntactic change of the number structure (i.e. 25 for 205) and lexical errors concerned one or more number elements and resulted in substitutions of numbers belonging to the same number-lexical class (i.e. 45 for 49). The evaluation was completed using card exercises. Nine cards were used. Each card had a different number of black circles corresponding to the numbers from 1 to 9. The examiner showed two cards and one algebraic symbol (“+” or “-“). The patient was then asked to make five additions and five subtractions. A spoken response was first required, followed by a written one. Finally, the patient had to choose the card with the number of circles corresponding to the result of the addition/subtraction. Only 2 responses out of 30 were correct.

Our patient underwent 1 month of intensive cognitive rehabilitation consisting of daily 2- h sessions, 5 days a week, doing traditional and computer assisted exercises to improve error awareness, re-learning and memory consolidation. Goals and therapeutic approaches were continuously modified and adapted to the patient’s cognitive performances. The rehabilitation program was carried out in order to improve the various aspects of the language system (semantic, phonological, syntactic). The use of technologies such as smartphones, tablets and social media were allowed and encouraged beyond the rehabilitative setting in order to stimulate his interactions abilities. Together with the language treatment, he also underwent a computerized rehabilitation program by means of the Rehacom Software (Hasomed GmbH Magdeburg, Germany), a widely utilized tool in cognitive rehabilitation.

For the rehabilitation of calculation, different types of exercises (pencil and paper or computer exercises) were alternatively administered. The treatment was aimed at stabilizing the processing of arithmetical signs “+”,”-“, “*”, “÷”, encouraging the implementation of support strategies such as using fingers to count or multiplication tables and consolidating and developing arithmetical calculation procedures.

He also underwent a month of intensive everyday one-hour sessions of motor, proprioceptive and balance rehabilitation treatment.

**Fig. 4:**
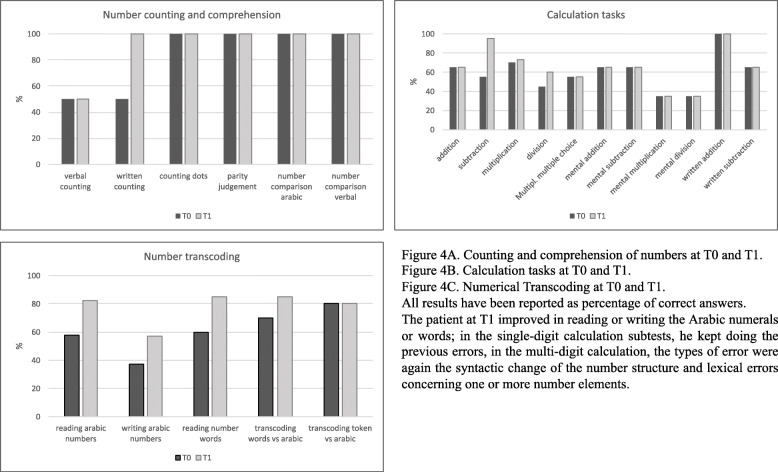
Results of the calculation tasks. All the results have been reported as a percentage of correct answers

After 1 month-long rehabilitation (T1) our patient presented an amelioration in all the domains (Table 1) except the calculations. Specifically, he improved his cognitive status in attention domain, language (minimal level of disability), executive functions, visuo-constructional praxis, memory, while unremarkable ameliorations were found in calculations. In the single-digit calculation subtests, he kept doing the previous errors, such as non-response or long latency times and difficulties in the correct interpretation of the arithmetical signs. He improved in reading and writing the Arabic numerals or words. In the multi-digit calculation, the types of error were again the syntactic change of the number structure and lexical errors concerning one or more number elements (Fig. 4).

Our patient, though unable to do elementary calculations, was able to play cards with moderate success. Specifically, he played a card game, in which the aim is to make simple additions to take all the cards present on the table. Given the weirdness of this situation, we tried to let him play with both Spanish and French cards. The Spanish cards, instead of numbers, are illustrated with sticks, cups, coins and swords, while the French cards with numbers associated with hearts, diamonds, flowers and spades. He could use the first or the latter, being able to easily calculate additions and compete fairly in the game, often winning against opponents. Given these abilities, we tried to make him play more complex games. In one of the proposed games the aim is to make additions, among the cards present on the table, up to obtain scores of 15; since he could fairly compete, we also tried a more complex card game, in which it is required to put all the 13 assigned cards in the correct order until they are finished, having to calculate the score at least up to 40. Our patient was able to play both of these games.

After discharge, the patient’s condition remained stable. He is now treated with Rituximab (according to Kim et al., 2013) [7].

## Discussion and conclusions

This subject demonstrated an improvement in all domains, except the calculations. To our knowledge, only two previous works reported calculations disorders in persons affected by AE. Loughan in 2016 [8] reported a case report of a 42-year-old male diagnosed with NMDAr encephalitis who complained reduced sustained attention, poor word retrieval, and daily forgetfulness 6 months after the diagnosis. The authors specifically investigated mathematic computations with the TMT-A and B, reporting intact performance respect to the premorbid status. In another paper [9], the authors reported the case of a 44-year-old mathematics teacher affected by NMDA encephalitis, that presented dyslexia, phonemic paraphasia, and dyscalculia. In this person all symptoms improved gradually after treatment and, 6 months later, she was able to go back to teach students. However, it is not specified which test was used to investigate the patient cognitive impairment.

A recent systematic review about cognitive outcomes following anti-NMDAr encephalitis in 109 treated patients (83.5% female, M_age_ = 22.5 years) documented high rates of neuropsychological dysfunction (in more than 75% of cases) [10]. The acute phase of recovery highlighted impairments of variable severity in overall cognitive domains, language, attention, working memory and visuo-spatial functions than in later phases of convalescence. Even if the enclosed papers don’t specifically consider calculation disturbances between the long-term deficits, the authors state that long-term outcomes revealed significant improvement (74.3%). As far as we know, our patient is the only one reported to have a permanent difficulty in computational skills.

Although in literature it is reported that deficits in executive control and episodic memory are less likely to be resolved [10], our patient presented an amelioration both in short-term and long-term cognitive abilities, but calculation abilities did not improve.

The low improvement in calculation may be due to a late diagnosis and to the severity of clinical presentation. Many studies investigate the role of different frequent variables as outcome predictors in patients with autoimmune encephalitis and the results are often conflicting [4, 5]. The authors found that predictors of good outcome are the lower severity of symptoms, assessed as no need for admission to ICU, and the prompt initiation of immunotherapy and tumor removal, whether appropriate; altered conscious state has been considered as a possible prognostic factor and it has been recognized to be correlated with poorer functional performance at a median of 12 months.

In fact, our patient was not rapidly diagnosed; on the contrary, initially a somatoform disorder was hypothesized and there was an important delay in the treatment that could explain the uncomplete recovery. Moreover, since the serious initial clinical conditions, the patient has needed an admission to ICU. Although he presented an effective improvement on several cognitive domains, he did not present a parallel improvement in calculation skills. When he went back at school, assistive modalities were provided to him to perform calculation tasks, such as a calculator and a personal computer.

The preserved ability to play card games where calculations are supposed to occur, could be explained taking into account the role of procedural memory. While in healthy conditions, the patient acquired and consolidated the skill to play with both French and Spanish cards and probably implicitly associated the aspect of the cards with their numerical value, being able to compare the value of the cards without making actual calculations while playing. This procedural skill is specific for the aspect of the cards since the patient was unable to perform calculations not only using numbers but also using printed dots, whose number (but not their aspect) could resemble the value of the cards. During skills acquisition the initial learning stages involve attention, many cortical areas and sensory-motor integration processes. When continuously repeated over weeks and months, the process becomes automatic and the activation of cortical areas is significantly reduced, involving mainly the motor cortex, posterior striatum and anterior cerebellum [11]. We assume that, following encephalitis, the cortical areas involved in performing new unlearned tasks, such as performing calculations in the context of a neuropsychological assessment, were significantly hampered. Conversely, previously learned skills related to card games were preserved.

Concluding, we suggest clinicians should consider a careful approach to determine the prognosis of AE because of the wide range of deficits, the need of prolonged treatment and the rate of long-term sequelae. In our patient the calculation disorder has proved to be the most relevant problem and the most difficult to treat. Future studies are needed to better understand this phenomenon and find the appropriate cognitive rehabilitative treatment.

## Data Availability

All data generated or analyzed during this study are included in this published article.

## Abbreviations

AE: Autoimmune encephalitis
NMDAr: N-methyl D-aspartate-receptor
ICU: Intensive Care Units
EEG: Electroencephalography
PET: Positron Emission Tomography
CT: Computed Tomography
MRI: Magnetic Resonance Imaging
CSF: Cerebro-spinal-fluid
MMSE: Mini-Mental State Examination
TMT-A: Trail Making Test part A
TMT-B: Trail Making Test part B
FAB: Frontal Assessment Battery
AAT: Aachener Aphasia Test
